# 

**Published:** 1904-07

**Authors:** 


### INDEX TO VOLUME XX.

A Case of Renal Calculus with Operation. By Jno. B. Thomas, M. D., Abilene, Texas.................................. 10
Albargin Joint Review with Personal Experience. By Prof. Dr. Seifert, Wurzburg ................................ 47
American Medico-Psychological Association—Sixty-first Annual Session, San Antonio, Texas, April 18, 19, 20, 1905.. 503
Bacteria and What We Know About Them. By J. M. Fort, M. D., Paris, Texas.................................. 487
Chronic Gastritis. By J. R. Hunter, M. D., Hornsby, Texas. 301
Compound Communicated Fracture of the Spinal Vertebrae, Extending from the Second Lumbar to the Base of the Sacrum, Exposing Cauda Equina—Operation and Recovery. By E. M. Albers, M. D., Pinos, Zac., Mexico................ 1
Digitalis and Veratrum in the Treatment of Pneumonia. By J. W. Carhart, M. D., Austin, Texas.................... 306
Dr. Osler's Little Joke....................................... 405
Etiology and Treatment of Puerperal Eclampsia. By Felix S. Martin, M. D., Beaumont, Texas................... 364
Ilio-Colitis.—Etiology and Treatment. By E. B. Parsons, M. D., Palestine, Texas.............................. 127
Legislative Conference at Austin November 14th and 15th.. 219
Medical and Sanitary Legislation.......................... 272
Meeting of the American International Congress on Tuberculosis, held Under the Auspices of the Universal Exposition of St. Louis, October 3, 4, and 5, 1904..............190, 214
Moccasin Bite Treated with Adrenalin Chloride. By R. Menger, M. D., San Antonio, Texas......................... 263
Modern Therapeutics and Pharmacy. By Frederick Hadra, M. D., San Antonio, Texas............................ 345
Multiplying Licensing Boards............................. 317
Notes on Pernicious Malarial Fever. By J. D. Jordan, M. D., Madisonville, Texas ..................................... 169
Now or Never ............................................... 58
On the Prevention of Typhoid and Malarial Fevers......... 3
Preventive Legislation in Forensic Medicine. By Clark Bell, Esq., LL. D., of the New York Bar...................... 85

Prohibition—Its Definition, Object, Feasibility and Consequences, with Other Considerations of the Subject from the Standpoint of a Physician. By C. H. Wilkinson, M. D., San Antonio, Texas.... 176
Proprietary Pharmacists and State Medical Association Journals.... 478
Rally at Houston.... 371
Rash Action of the House of Delegates.... 473
Reminiscences of an Old Doctor. By J. M. Fort, M. D., Paris, Texas.... 357
Sentiment and Science. By F. E. Daniel, M. D.... 429
Some Remarks on the Operation for Hernia. By T. J. Bennett, M. D., Austin, Texas.... 259
Strangulated Hernia in Man of Eighty-five—Operation. By Geo. Harwood, M. D., Johnson City, Texas.... 369
The Care of the Insane in Texas. By M. L. Graves, M. D.... 387
The Chicago Octopus: The Journal American Medical Association.... 497
The Great International Tuberculosis Congress.... 60
The Great International Congress on Tuberculosis, St. Louis, October 3, 4 and 5.... 103
The Medical Treatment and Management of an Attack of Appendicitis—with a Review of Some of the Literature of the Subject, both Old and New. By Boyd Cornick, M. D., San Angelo, Texas.... 90
The Medical Profession—A Contributory Factor to the Death Rate of Consumptives. By C. H. Wilkinson, M. D., San Antonio, Texas.... 43
The Practice of Medicine.... 280
The Prohibition Craze in Texas.... 235
The Two Ends of My Experience with the Lymph Compound. By C. N. Palmer, M. D., Lockport, N. Y.... 400
The Vaginal Route in Pelvic Surgery. By O. L. Norseworthy, M. D., Houston, Texas.... 131
The Woman's Peril; the Man's Sin. Letter from Dr. Josephine Kingsley.... 51
Thirty-seventh Annual Meeting, State Medical Association of Texas, Houston, Texas, April 24, 25, 26, 27, 28, 1905.... 450
Treatment of Typhoid Fever. By Robert M. Sterrett, M. D., New York, N. Y.... 136

